# Fall-related Injuries in Older Adults and Medications Prescribed within 30 Days Prior to the Injury

**DOI:** 10.1093/geroni/igab046.2899

**Published:** 2021-12-17

**Authors:** Yu Ming, Aleksandra Zecevic, Richard Booth, Susan Hunter, Andrew Johnson, Rommel Tirona

**Affiliations:** 1 St. Michael's Hospital, London, Ontario, Canada; 2 Western University, London, Ontario, Canada

## Abstract

Fall-related injuries in older adults have serious consequences both for individuals and the public health care system. The purpose of this study was to identify medication classes prescribed within 30 days prior to the injury that were associated with fall-related injuries in older adults. This population-based, case-control study used secondary administrative health care data in Ontario, Canada. The cases were older adults, aged 66 years and older, who visited an emergency department for a fall-related injury. Controls were extracted from the Registered Person Database, and matched by same age, sex and residence area. Medication classes prescribed to both groups were recorded and logistic regression was conducted to examine the association between medications and fall-related injury. The case group included 255,270 older adults who experienced a fall-related injury over the five-year period (2010-2014). After adjustment for sex, age group, residence area, income level and number of medications prescribed, psychotropic medications (i.e., opioids, anti-epileptics, anti-Parkinson’s drugs, and antidepressants), drugs for treatment of constipation, infection and benign prostatic hyperplasia, antithrombotic agents, statins and bronchodilators were identified to be related to increased risk of fall-related injuries. In addition to medications already on the list of fall-risk increasing drugs or FRIDs, this study uncovered that drugs for benign prostatic hyperplasia, cephalosporins, biphosphates and bronchodilators increased the risk of fall-related injury in older adults. Well-designed prospective cohort studies considering prescription indication and drug-drug interactions are needed to provide more convincing evidence on medications that may be associated with increased risks of fall-related injury in older adults.

